# Evaluating the diagnostic accuracy of dynamic CT transmural sign for T staging of gastric cancer compared to conventional CT criteria

**DOI:** 10.1186/s12880-025-01728-8

**Published:** 2025-05-21

**Authors:** Bona Lee, Dong Jin Chung, Min Gang Jo, Seung Eun Lee, Tae Jung Kim, Sung Geun Kim, Dae Young Cheung

**Affiliations:** 1 Department of Radiology, College of Medicine, Yeouido Saint Mary’s Hospital, The Catholic University of Korea, Seoul, Republic of Korea; 2 Department of Radiology, Seoul Saint Mary’s Hospital, College of Medicine, The Catholic University of Korea, Seoul, Republic of Korea; 3 Department of Hospital Pathology, College of Medicine, Yeouido Saint Mary’s Hospital, The Catholic University of Korea, Seoul, Republic of Korea; 4 Division of Gastrointestinal Surgery, Department of General Surgery, College of Medicine, Yeouido Saint Mary’s Hospital, The Catholic University of Korea, Seoul, Republic of Korea; 5 Division of Gastroenterology, Department of Internal Medicine, Yeouido Saint Mary’s Hospital, College of Medicine, The Catholic University of Korea, Seoul, Republic of Korea

**Keywords:** Gastric cancer, Staging, Computed tomography, Histopathology

## Abstract

**Background:**

To retrospectively evaluate the diagnostic performance of pre-procedural multidetector computed tomography (MDCT) using dynamic CT transmural (CTTM) criteria for T staging of gastric cancer.

**Methods:**

This retrospective study enrolled 116 patients who underwent three-phase dynamic MDCT and subsequently received endoscopic treatment or surgery. The positive CTTM sign was defined as a new diagnostic criterion for this study. Two radiologists independently reviewed the CT images, categorizing patients into five groups: T1b sm1, T1b sm2/3, T2, T3, and T4. The diagnostic performance of the new criterion was assessed against pathological results as the gold standard. Sensitivity, specificity, accuracy, and positive and negative predictive values for each T stage were compared to those of conventional CT.

**Results:**

Dynamic CTTM criteria demonstrated higher overall diagnostic accuracy (Reviewer 1: 88.9%, Reviewer 2: 91.4%) for T staging compared to conventional CT criteria (75.9%). Reviewer 1 accurately staged T1b in 85.3% of patients, T2 in 87.7%, T3 in 94.1%, and T4 in 98.3%. Reviewer 2 achieved T1b accuracy of 87.9%, T2 of 89.2%, T3 of 94.9%, and T4 of 98.3%. The dynamic CTTM criteria effectively subdivided T1b into T1b sm1 (87.2% and 88.1%) and T1b sm2/3 (84.6% and 86.2%). Dynamic CTTM criteria exhibited higher under-staging rates, while conventional criteria showed higher over-staging rates.

**Conclusions:**

Dynamic CTTM criteria demonstrated superior accuracy in T staging of gastric cancer and reasonable effectiveness in subdividing T1b stages into T1b sm1 and T1b sm2/3.

## Background

The depth of tumor invasion is a crucial prognostic factor in gastric cancer [1, 2]. With the increasing use of endoscopic mucosal resection or submucosal dissection (EMR or ESD) for treating early gastric cancer (EGC), there is a pressing need for more accurate staging. Differentiating between T1b and T2, or even between T1b sm1 and T1b sm2/3, is essential for both endoscopists and surgeons to determine optimal treatment options [3, 4]. Standard imaging modalities for preoperative (TNM) staging include endoscopic ultrasound (US) for assessing tumor depth and computed tomography (CT) for evaluating lymph nodes and distant metastasis. However, recent studies indicate that the detection of T stage shows no significant difference between CT and endoscopic US [5, 6]. Practically, CT scans are often preferred over endoscopic ultrasound in real-world scenarios, highlighting the importance of accurate disease staging through CT. Most previous studies have employed conventional CT criteria based on the appearance of normal gastric walls, which are typically seen as three layers on contrast-enhanced CT images: (1) an inner mucosal layer with marked enhancement, (2) a submucosal layer with low attenuation, and (3) an outer muscular-serosal layer with slightly higher attenuation [7]. In practice, the normal gastric wall exhibits varying layered patterns on contrast-enhanced CT based on luminal distension or the specific location in the stomach. Under gaseous distension, the gastric wall may present single- or double-layered patterns, likely due to the stretching of the submucosal layer. Additionally, with an average thickness of 4.8 mm under gaseous distension, accurately distinguishing the depth of tumor invasion can be challenging [8]. To address the limitations of conventional CT criteria, we propose reducing the number of visible layers from three to two, focusing on the inner layer with high density and the outer layer with low density. Prior research has suggested that incorporating the arterial phase can enhance assessment accuracy for invasion depth [9]. Thus, we introduce a new staging criterion termed the “dynamic CT transmural (CTTM) sign,” which evaluates the depth and progression of enhancement through each of the three phases of dynamic CT. This pattern-based approach provides an indirect assessment of invasion depth. Using the dynamic CTTM sign, we retrospectively evaluated the diagnostic performance of the new CT criteria for determining T staging in patients with gastric cancer, as outlined in the 8th edition of the AJCC manual. Further, we compared the diagnostic utility of dynamic CTTM criteria with conventional staging methods.

## Methods

This single-center, retrospective study was approved by the Institutional Review Board of the Ethics Committee. The requirement for informed consent was waived due to the study’s retrospective design.

### Patient participation

**Fig. 1:**
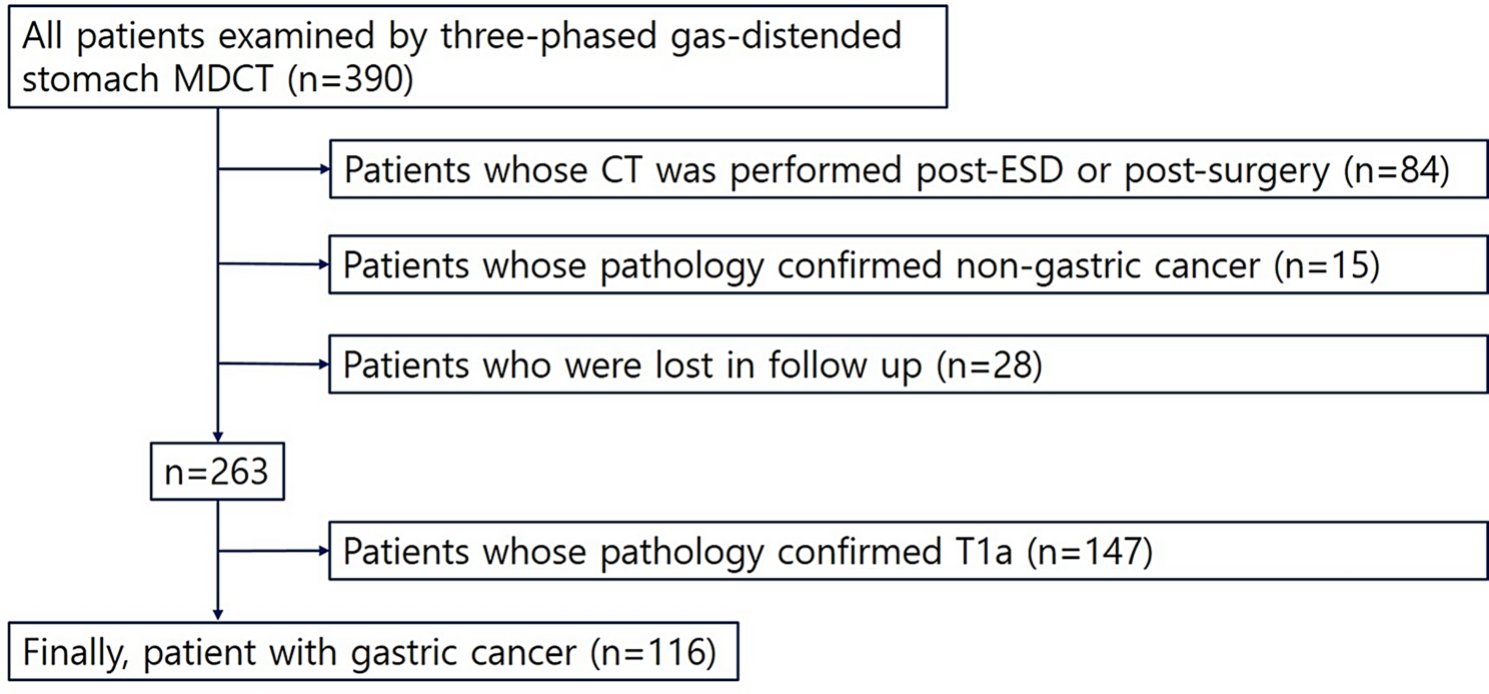
Flow chart of patient inclusion and exclusion criteria; ESD, endoscopic submucosal dissection

Between July 2014 and June 2020, a total of 390 patients who underwent three-phase gas-distended stomach multidetector computed tomography (MDCT) were included in this study. The inclusion criteria were as follows: (a) pre-operative endoscopic biopsy results confirming gastric adenocarcinoma and (b) patients who underwent endoscopic dissection or surgery. The exclusion criteria included: (a) patients whose CT scans were performed after endoscopic submucosal dissection (ESD) or surgery; (b) patients with pathology confirming non-gastric cancer; (c) patients lost to follow-up; and (d) patients with pathology confirming T1a. Non-malignant gastric wall thickening observed on CT and T1a tumors were excluded from the analysis because they are often not visible on CT scans, which can significantly impact the accuracy of our results. Given their substantial presence (147 out of 390) in the patient population, including them could distort the statistical outcomes. Therefore, we focused on more advanced gastric cancers to ensure the validity and reliability of our findings. We acknowledge that the retrospective design of the study introduces potential biases, such as selection bias in patient enrollment. To mitigate these biases, we employed strict inclusion and exclusion criteria, ensuring that only patients meeting the defined criteria were included in the analysis. This approach helps to improve the validity of our findings. Ultimately, 116 patients with gastric cancer were enrolled in the study (85 males and 31 females), with a mean age of 70.0 years (range: 43–94). Details of patient inclusion and exclusion are presented in Fig. 1, and patient characteristics are summarized in Table 1.

**Table 1:** Patient characteristics

Characteristics	Patients (*n* = 116) (%)
Age (years)	
Mean ± SD	70.0 ± 11.2
Median (range)	71.0 (43–94)
Sex	
Male	85 (73.3)
Female	31 (26.7)
Pathologic T staging	116
T1b	45
T1b sm1	10
T1b sm2/3	35
T2	20
T3	25
T4	26
Tumor location on longitudinal axis	
Cardia	8 (6.8)
Fundus	2 (1.7)
Body	42 (36.2)
Angle	12 (10.2)
Antrum	36 (30.5)
More than two aspects	16 (13.6)
Cell type	
Differentiated	44 (38.0)
Undifferentiated	72 (62.0)

### MDCT image acquisition

Before undergoing CT, butylscopolamine bromide (Buscopan; Boehringer Ingelheim, Germany) was administered intramuscularly to patients to relax the gastric wall and minimize bowel peristalsis. Subsequently, two packs of gas-producing crystals (totaling 8 g) mixed with minimal water (< 10 mL) were given orally to distend the stomach. A dual-source CT scanner (Somatom Definition Flash; Siemens Healthcare, Erlangen, Germany) was utilized, employing a dual-energy protocol (collimation: 128 × 0.6 mm; pitch: 0.6; rotation time: 0.5 s; reconstruction interval: 3 mm) with a tube voltage of 120 kVp and automated attenuation-based tube current modulation (Care Dose 4D; Siemens Healthcare).

MDCT was performed in the arterial, portal, and delayed phases after intravenous injection of Iohexol (Iobrix 300; Taejoon Pharm, Kyungkido, Republic of Korea) at rates of 4 mL/sec through the brachial peripheral vein, followed by a 20 mL saline flush. To minimize the differences in the degree of contrast enhancement in the stomach, we placed a region of interest (ROI) on the descending aorta. We obtained the arterial phase images 15 s after the enhancement reached 100 HU. Subsequently, we acquired the portal phase images 20 s later and the delayed phase images 100 s after that. On average, the images were obtained at 40 s (for the arterial phase), 70 s (for the portal phase), and 150 s (for the delayed phase). Pre-contrast CT was not conducted. Scans were obtained with the patients in two positions: the left lateral decubitus position for the arterial and portal phases, and the supine position for the venous phase. The mean volumetric CT dose index was 21.8 mGy ± 4.3 (range: 16.7–32.6 mGy), and the mean dose-length product was 808.2 mGy·cm ± 49.8 (range: 650–1211 mGy·cm).

### Definition of dynamic CTTM sign

**Fig. 2:**
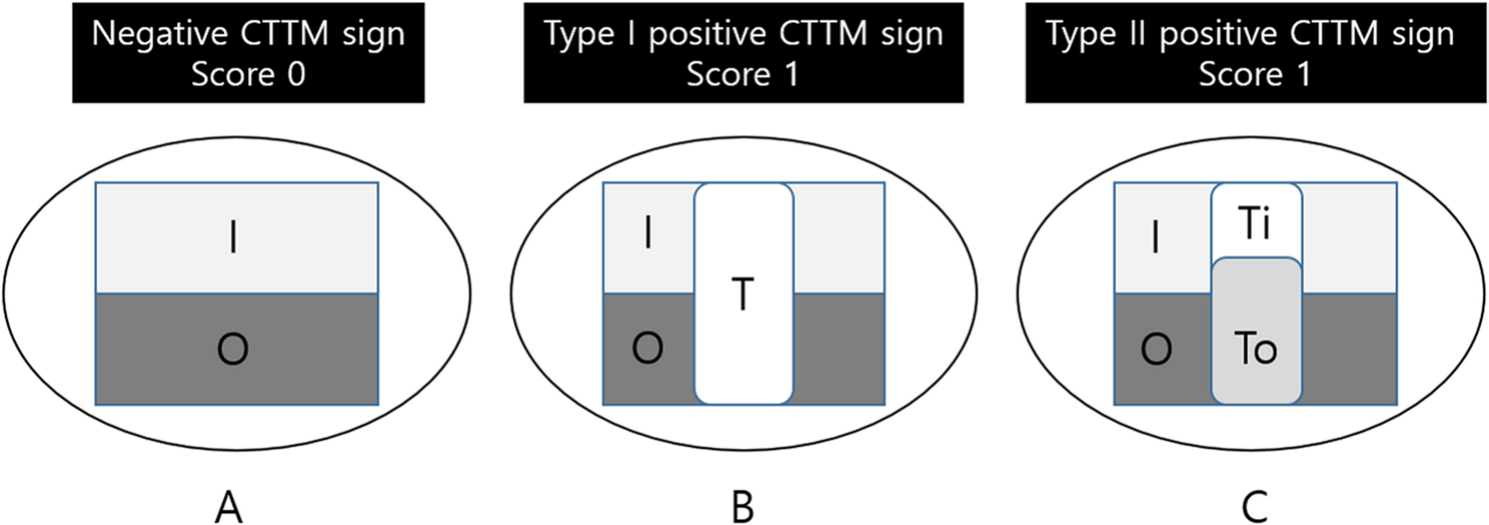
Diagram of Positive Dynamic CTTM. I: inner wall, O: outer wall, T: tumor, Ti: tumor inner, T0: tumor outer, Score 0: negative CTTM sign, Score 1: positive CTTM sign. (**A**) The typical pattern of normal gastric wall enhancement presents a thin, two-layer appearance characterized by an enhancing inner layer and a non-enhancing outer layer. However, this two-layer enhancement does not correlate with the anatomical five layers of the stomach. (**B**) Type I Dynamic CTTM Sign. Both the inner and outer layers of the mass demonstrate uniform enhancement, indicating a single transmural enhancement pattern. (**C**) Type II Dynamic CTTM Sign. The inner layer of the mass displays contrast enhancement, while the outer layer appears thicker with mild contrast enhancement compared to the typical outer gastric layer, resulting in a double-layered transmural enhancement

A transmural enhancing tumor, which involves the entire gastric wall, can be classified into two distinct types based on dynamic computed tomography (CT) imaging. We propose the term “positive dynamic CT transmural (CTTM) sign,” as depicted in Fig. 2. Type I condition refers to the homogeneous single-layer enhancement of the entire lesion wall. Type II condition is characterized by a two-layer enhancement pattern. The inner layer demonstrates significant contrast enhancement, while the outer layer is thicker and shows mild contrast enhancement in comparison to the normal outer gastric layer, resulting in a double-layered transmural enhancement. Although the entire gastric wall may exhibit enhancement as a single layer in the delayed phase, resembling a positive CTTM sign, it is defined as a positive sign when the enhancement differs from that of the surrounding normal gastric wall (either hypo- or hyperattenuated). It is essential to highlight that the two-layer enhancement observed in the dynamic CTTM sign on CT scans does not correspond to the anatomical five layers of the stomach. This pattern-based approach differs from the conventional CT criteria for assessing the depth of invasion. Consequently, the primary objective of this study is to evaluate the depth of invasion by analyzing the contrast enhancement patterns presented in dynamic CT images.

### Image analysis of T staging by new and conventional criteria

**Fig. 3:**
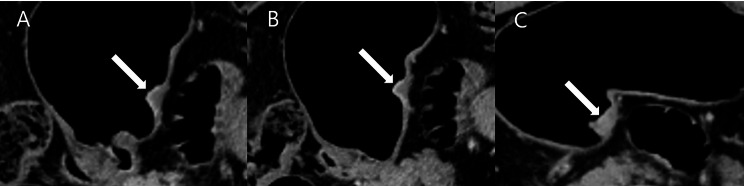
A patient with differentiated T1b sm2/3 stomach cancer. **A** and **B**. In the arterial and portal phases, the lesion (arrow) shows wall thickening characterized by double-layered enhancement, comprising an inner enhancing layer and an outer non-enhancing layer. Both phases demonstrate an outer layer with a thin enhancement pattern resembling the normal gastric outer layer, indicative of a negative CT transmural sign. **C**. In the delayed phase, the thickened gastric wall exhibits transmural enhancement as a single layer (arrow), representing a type I positive dynamic CTTM sign. The CTTM pattern is classified as 0-0-1

**Fig. 4:**
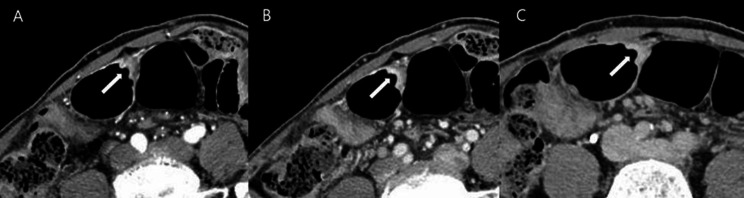
A patient with differentiated T2 stomach cancer. (**A**) In the arterial phase, the lesion (arrow) shows a double-layered wall with a non-enhancing outer layer, indicative of a negative dynamic CTTM sign. (**B**) In the portal phase, the lesion demonstrates a type II positive dynamic CTTM sign due to the thickened and higher enhancement of the outer low-density layer (arrow) compared to the normal gastric outer layer. (**C**) In the delayed phase, the lesion shows single-layer transmural enhancement (arrow), representing a type I positive CT transmural sign. The CTTM pattern is classified as 0-1-1

**Fig. 5:**
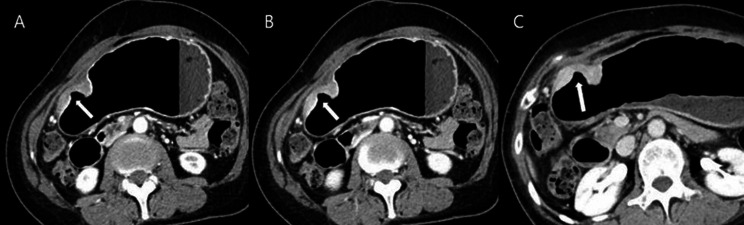
A patient with differentiated T3 stomach cancer. **A** In the arterial phase, a homogeneously enhancing mass (arrow) is noted on the anterior wall of the gastric angle. The lesion exhibits single-layer transmural enhancement, consistent with the type I positive dynamic CTTM sign. **B** and **C** In the portal and delayed phases, the lesion shows pronounced transmural enhancement (arrow), confirming the type I positive dynamic CTTM sign. The CTTM pattern is classified as 1-1-1

**Fig. 6:**
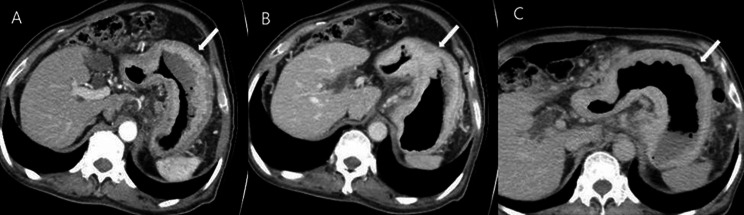
A patient with poorly cohesive type T4a stomach cancer. **A** and **B** In the arterial and portal phases, diffuse thickening of the gastric wall along the greater curvature of the gastric body is observed. This thickening exhibits a double-layered appearance, indicative of a type II positive dynamic CTTM sign, resulting from the thickened and enhanced outer low-density layer (arrow) compared to the normal gastric outer layer. **C** In the delayed phase, the lesion displays homogeneous transmural enhancement with perigastric fat strands, characterized as a type I positive CT transmural sign (arrow). The CTTM pattern is classified as 1-1-1

Two radiologists (reviewer 1 with 12 years and reviewer 2 with 26 years of experience in interpreting the abdominal CT images) blinded to pathologic results reviewed the pre-procedural CT images independently. They categorized all 116 patients into five groups based on the number of positive dynamic CT transmural (CTTM) signs observed in the arterial-portal-delayed phase. Coronal and sagittal reformatted images, along with axial images, were also used in the analysis. The CTTM sign was represented as negative with a score of 0 and as positive with a score of 1. For T1b sm2/3, the pattern is 0-0-1, indicating a positive sign only in the delayed phase (Fig. 3). For T2, the pattern is 0-1-1, indicating positivity in both the portal and delayed phases (Fig. 4). T3 shows a pattern of 1-1-1, indicating a positive sign in all phases. Lastly, T4 also presents a pattern of 1-1-1, reflecting positivity across all phases with involvement of perigastric fat strands (Figs. 5 and 6). Patients whose supposed cancer was not visible at the endoscopically confirmed location on the CT images were classified as T1a and were ultimately excluded in this study. To compare the diagnostic performance of the new and conventional T staging, the reviewers retrospectively re-analyzed the portal phase images of MDCT using the conventional criteria. The conventional method divides the group into four categories: T1 without substaging, T2, T3, and T4. The conventional criteria follow the imaging guidelines based on the 8th edition of the American Joint Committee on Cancer (AJCC). The conventional CT criteria defines focal wall gthickening and enhancement localized in the inner and/or middle layers as T1, focal wall thickening and enhancement equal to or more than half of the gastric wall thickness as T2, and transmural enhancing tumor involving the entire gastric wall without perigastric fat infiltration as T3 cancer [10–12]. In both criteria, we analyzed the images using all phases of CT scans for analysis. Details of new MDCT and conventional criteria are described in Table 2.

**Table 2:** New MDCT imaging criteria using CT transmural sign and conventional MDCT imaging criteria for T staging of gastric cancers

		New MDCT imaging criteria and CTTM patterns	Conventional MDCT imaging criteria
T staging	T1b sm1	Negative dynamic CTTM sign in all phases (0-0-0)	Abnormally enhancing mass or wall thickening less than half of the gastric wall thickness
	T1b sm2 or sm3	Positive dynamic CTTM sign only in the delayed phase (0-0-1)	
	T2	Positive dynamic CTTM sign in the portal and delayed phases (0-1-1)	Abnormally enhancing mass or wall thickening equal to or more than half of the gastric wall thickness but with maintenance of the thin most outer layer
	T3	Positive dynamic CTTM sign in all phases without perigastric fat strands (1-1-1)	Transmural enhancing tumor involving the entire gastric wall without perigastric fat infiltration or with a few perigastric fat stranding below the adjacent perigastric vessels
	T4	Positive dynamic CTTM sign in all phases with perigastric fat strands (1-1-1)	Transmural tumor involving the entire gastric wall with irregular/nodular outer border and/or fat infiltration invading beyond the adjacent perigastric vessels. (T4b, if there is adjacent organ invasion by mass itself and/or perigastric fat infiltration)

### Surgical and pathological evaluation

Among the 116 patients, surgical resection was performed in 112 cases, while endoscopic submucosal dissection (ESD) was conducted in 4 cases. Pathological data were utilized as the reference standard for T staging in this study. All gastric cancers were categorized into two main groups: differentiated and undifferentiated types [11, 13]. The undifferentiated category included poorly differentiated tubular adenocarcinoma, signet-ring cell carcinoma (cohesive type), mucinous adenocarcinoma, and lymphoepithelioma-like carcinoma [11, 13, 14]. Any mixed patterns that included one of the undifferentiated types were classified as undifferentiated.

### Statistical analysis

For each reviewer, the accuracy, sensitivity, specificity, as well as the proportions of over-staging and under-staging for T staging based on both conventional and new CT criteria were calculated. Inter-reader agreement was assessed using weighted Kappa statistics with linear weights, interpreted as follows: poor (< 0.20), fair (0.20–0.39), moderate (0.40–0.59), substantial (0.60–0.79), and almost perfect (> 0.80). All statistical analyses were conducted using commercially available statistical software, SAS version 9.4 (SAS Institute, Cary, NC, USA). Differences were considered statistically significant when the P-value was less than 0.05.

## Results

### Histopathologic results

All patients had histopathologically confirmed gastric adenocarcinoma. Among them, two patients had two separate gastric carcinomas with different pathological T staging following surgery. One patient had lesions staged as pT3 and pT2, while the other had pT1b sm2 and pT1a staged lesions. For the purposes of T staging analysis and evaluation of diagnostic performance, only the higher-stage lesion was analyzed in each case. Post-ESD or post-surgical pathological results were categorized into five groups: pT1b sm1, pT1b sm2/3, pT2, pT3, and pT4a/b. Of the 116 patients, 10 were confirmed with pT1b sm1, 35 with pT1b sm2/3, and 45 with pT1. Additionally, 20 patients were diagnosed with pT2, 25 with pT3, and 26 with pT4a or greater. In terms of histological classification, 44 cancers were categorized as differentiated, while 72 were classified as undifferentiated.

### Diagnostic performance of dynamic CTTM criteria for T staging

Reviewers 1 and 2 demonstrated overall T staging accuracies of 88.8% (103/116) and 91.4% (106/116), respectively, when using the new dynamic CT transmural (CTTM) criteria. In contrast, both reviewers showed the same accuracy of 75.9% based on conventional criteria. With the dynamic CTTM criteria, the over-staging rates were 1.7% (2/116) for both reviewers, while the under-staging rates were 9.5% (11/116) for Reviewer 1 and 6.9% (8/116) for Reviewer 2. In comparison, conventional criteria displayed higher over-staging rates of 17.2% for both reviewers for T staging (Table 3). The Kappa agreement between reviewers was 0.59 (95% confidence interval: 0.37–0.77), indicating moderate agreement; this suggests that there is a certain level of consistency in the application of the dynamic CTTM criteria, but the degree of variability among reviewers should be considered, as it may affect the reliability in clinical practice. The new dynamic CTTM criteria provided better diagnostic accuracy for T1b, T2, and T3 staging compared to conventional criteria. Reviewer 1 achieved staging accuracies of 85.3% (*p* = 0.048) for T1b, 87.7% (*p* = 0.005) for T2, 94.1% (*p* = 0.004) for T3, and 98.3% (*p* = 0.004) for T4. Reviewer 2 had corresponding accuracies of 87.9% (*p* = 0.048) for T1b, 89.2% (*p* = 0.001) for T2, 94.9% (*p* = 0.001) for T3, and 98.3% (*p* = 0.001) for T4. Detailed diagnostic performance results, including sensitivity, specificity, positive predictive value, and negative predictive value for each T stage by each reviewer, are provided in Table 4. There was no statistically significant difference in diagnostic accuracy between the two criteria for T4 staging.

**Table 3:** Diagnostic performance of dynamic CTTM sign criteria for T staging

MDCT criteria	Reviewer 1			Reviewer 2		
	Accuracy (%)	Over-staging (%)	Under-staging (%)	Accuracy (%)	Over-staging (%)	Under-staging (%)
Dynamic CTTM	88.8 (103/116)	1.7 (2/116)	9.5 (11/116)	91.4 (106/116)	1.7 (2/116)	6.9 (8/116)
Dynamic CTTM including substaging of T1b	85.3 (99/116)	3.5 (4/116)	11.2 (13/116)	87.9 (102/116)	3.5 (4/116)	8.6(10/116)
Conventional	75.9 (88/116)	17.2 (20/116)	6.9 (8/116)	75.9 (88/116)	17.2 (20/116)	6.9 (8/116)

Values are presented with raw numbers in parentheses

**Table 4:** Sensitivity, specificity, positive predictive value of dynamic CTTM criteria

Pathology	Reviewer 1					Reviewer 2				
	Sensitivity	Specificity	Accuracy	PPV	NPV	Sensitivity	Specificity	Accuracy	PPV	NPV
T1b including substaging	86.7 (0.008)	79.7 (0.046)	85.3 (0.048)	75.9 (0.15)	87.9 (0.01)	87.7 (0.008)	82.5 (0.157)	87.9 (0.048)	79.2 (0.400)	89.0 (0.009)
T2	84.0 (0.059)	86.9 (0.035)	87.7 (0.005)	82.5 (0.015)	87.1 (0.037)	85.0 (0.02)	88.9 (0.011)	89.2 (0.001)	85.3 (0.006)	89.0 (0.013)
T3	84.0 (0.564)	96.8 (0.003)	94.1 (0.004)	87.5 (0.003)	90/94 (0.408)	88.0 (0.157)	96.8 (0.003)	94.9 (0.001)	88.0 (0.002)	96.8 (0.108)
T4	92.3 (NA)	100.0 (NA)	98.3 (0.046)	100.0 (0.317)	97.9 (0.414)	92.3 (NA)	100 (NA)	98.3 (0.046)	100 (0.317)	97.9 (0.414)

Data are presented as percentages, with 95% confidence intervals in parentheses

### Diagnostic performance of dynamic CTTM criteria for substaging of T1b gastric cancer

In T1b staging (with T1a excluded from this study), the dynamic CTTM criteria demonstrated higher diagnostic accuracy compared to the conventional criteria (86.1% vs. 78.3%, *p* = 0.04). The dynamic CTTM criteria also exhibited excellent diagnostic performance in subdividing T1b stages into T1b sm1 and T1b sm2/3. The diagnostic accuracies for reviewers 1 and 2 were 87.2% and 88.1% for T1b sm1 staging, respectively, and 84.6% and 86.2% for T1b sm2/3 staging, respectively. Since the conventional CT criteria do not subdivide T1 staging, this analysis was not applicable (Table 4).

### T staging accuracy in differentiated versus undifferentiated gastric cancer

For differentiated type gastric cancer, the overall diagnostic accuracy for T staging was 89.1% (40/45) for Reviewer 1 and 90.9% (41/45) for Reviewer 2. For undifferentiated type gastric cancer, the accuracies were 84.2% (61/73) for Reviewer 1 and 85.3% (62/73) for Reviewer 2. There was no statistically significant impact of histological type (differentiated versus undifferentiated) on the diagnostic accuracy of T staging for either reviewer (Table 5).

**Table 5:** Diagnostic performance of dynamic CTTM sign criteria for substaging of T1b cancer and histologic grading

Pathology	Dynamic CTTM sign						Conventional CT					
	Reviewer 1			Reviewer 2			Reviewer 1			Reviewer 2		
	Accuracy	Over-staging	Under-staging	Accuracy	Over-staging	Under-staging	Accuracy	Over-staging	Under-staging	Accuracy	Over-staging	Under-staging
T1b (*n* = 45)	85.3	4.4 (2/45)		87.9	4.4 (2/45)		75.9	15.5 (7/45)		78.3	6.7 (3/45)	
T1b sm1 (*n* = 10)	87.2			88.1								
T1b sm2/3 (*n* = 35)	84.6	5.7 (2/35)	5.7 (2/35)	86.2	86.2 (3/35)	5.7 (2/35)						
Histologic grading												
Differentiated	89.1	2.2 (1/44)	4.5 (2/44)	90.9 (40/44)	2.3 (1/44)	6.8 (3/44)	75 (33/44)	22.7 (10/44)	2.3 (1/44)	72.7 (32/44)	22.7 (10/44)	4.5 (2/44)
Undifferentiated	84.2	4.2 (3/72)	11.1 (8/72)	85.3	13.9 (10/72)	9.7 (7/72)	81.9	4.2 (3/72)	13.9 (10/72)	73.6	13.9 (10/72)	12.5 (9/72)

Values are presented with raw numbers in parentheses

## Discussion

In Korea, extended absolute indications for endoscopic submucosal dissection (ESD) include: (1) intramucosal differentiated adenocarcinoma without ulcers, regardless of lesion size; (2) intramucosal differentiated adenocarcinoma of 3.0 cm or less even with an ulcer; and (3) lesions smaller than 3.0 cm with submucosal infiltration depth of 500 μm (sm1) [15]. This study aimed to enhance the accuracy of T staging for gastric cancer and to subdivide early gastric cancer (EGC) stages into T1b sm1 and T1b sm2/3 using a novel CT diagnostic criterion. This new criterion is clinically significant for distinguishing T1b sm2/3 from T2, ultimately guiding optimal treatment decisions.

Historically, transmural enhancement has been associated primarily with gastric cancer stages T3 and beyond. However, our study reveals that transmural signs can also begin at T1b sm2/3. We defined T1b sm2/3 cancer as exhibiting a positive dynamic CTTM sign solely in the delayed phase of three-phase dynamic CT. This definition is supported by the observation that gastric cancer with robust enhancement progresses from the late arterial phase through the delayed phase, starting from the inner mucosal layer to the outer margins of the tumor. Therefore, well-enhancing T1b sm2/3 cancers would show enhancement in both the mucosal and submucosal layers during the delayed phase [16].

Additionally, tumors invading the submucosal layer are expected to enhance adjacent normal muscular and serosal layers. The mechanism remains unclear; however, theories suggest that malignant tumors, such as gastric cancer, develop at sites of chronic injury, resulting in invasive properties and adjacent tumor budding [17–19]. Based on this theory, we hypothesized that malignant lesions in the submucosal layer might restrict the diffusion of contrast in adjacent muscular and serosal layers due to cancer-related fibrosis and tumor budding. Thus, T1b sm2/3 cancers exhibit a positive CT transmural sign in the delayed phase according to our new diagnostic criterion.

As the tumor stage advances, neovascularity and cancer-related fibrosis become more extensive, leading to earlier and stronger enhancement. Consequently, T2 and T3/T4 cancers are defined as having positive CT transmural signs in the portal and delayed phases and in all phases, respectively.

Conventional CT criteria evaluate only the enhancing portions of lesions regarding depth of invasion and assess transmural enhancement solely in portal and delayed phase images. This approach presents two significant pitfalls: First, since transmural-enhancing lesions are classified as T3 or greater, T2 gastric cancers may be overstaged. Second, the conventional criteria do not recognize the type II dynamic CTTM sign as a valuable indicator, potentially leading to understaging of T3 gastric cancers as T2. In contrast, the new MDCT staging criteria consider both transmural (type I dynamic CTTM sign) and double-layered (type II dynamic CTTM sign) enhancing lesions as positive indicators of depth of invasion in gastric cancer and employ all three phases of dynamic imaging, including the arterial phase. These advancements contribute to improved diagnostic accuracy for overall T staging and T1b substaging.

Prior studies have indicated that increased stromal vascularity correlates with good differentiation in gastric carcinomas [14, 20]. Conversely, undifferentiated types exhibit decreased vascular structure compared to differentiated types, leading to delayed contrast enhancement. Research suggests that differentiated or mixed type gastric cancers with abundant neovascularity display the greatest enhancement in arterial or portal phases, while undifferentiated types tend to show stronger enhancement in delayed phases due to immature fibrosis [21]. However, our study found no difference in diagnostic performance of the new CT criterion between differentiated and undifferentiated type gastric cancers. We cannot yet predict how the improved T staging may influence treatment choices (e.g., endoscopic resection vs. surgery). Further research and advancements in CT technology are needed to differentiate between T1a, T1b sm1, and T1b sm2/3.

This study has limitations. First, comparing the density between the outer low-attenuating layer and the normal gastric outer layer in type II positive dynamic CTTM may be challenging due to the thinness of the outer layer in some cases. To achieve a more objective analysis of type II positive dynamic CTTM and increase interobserver agreement, we believe that incorporating texture analysis would be beneficial [22]. This approach would enable quantification of tissue characteristics, enhancing assessment accuracy and providing deeper insights into underlying pathological processes. Second, excluding T1a lesions and non-gastric cancers may limit the applicability of our findings and introduce selection bias. Future studies should include a broader range of gastric cancer cases for better generalizability. Third, the lack of discussion on lymph node metastases is a notable gap, particularly as the risk increases in T1b tumors. Lymph node involvement is critical for staging and influences treatment decisions, such as surgical intervention and adjuvant therapy. Addressing this aspect could provide valuable insights into prognosis and recurrence risk, highlighting the need for personalized treatment strategies. Fourth, this is a single-center observational study with a relatively small sample size. We acknowledge that a larger sample size would enhance the robustness of the results; however, we believe that our strict inclusion and exclusion criteria allow for meaningful conclusions within the studied population. Future research with a larger, multi-center cohort would be valuable for confirming our findings across broader populations.

## Conclusions

In conclusion, the new CT criteria utilizing the dynamic CTTM sign demonstrated higher accuracy in differentiating T stages of gastric cancer, particularly in T1b, T2, and T3 stages, and provided meaningful diagnostic performance in subdividing T1b gastric cancer into T1b sm1 and T1b sm2/3.

## Data Availability

The data that support the findings of this study are not openly available due to reasons of sensitivity and are available from the corresponding author upon reasonable request. Data are located in controlled access data storage at The Catholic University Institute.

## Abbreviations

CTTM: CT transmural
EMR: Endoscopic mucosal resection
ESD: Endoscopic submucosal dissection
MDCT: Multidetector computed tomography (MDCT)
US: Ultrasound
EGC: Early gastric cancer
